# Diagnostic Accuracy and Acceptability of the Primary Care Posttraumatic Stress Disorder Screen for the *Diagnostic and Statistical Manual of Mental Disorders* (Fifth Edition) Among US Veterans

**DOI:** 10.1001/jamanetworkopen.2020.36733

**Published:** 2021-02-04

**Authors:** Michelle J. Bovin, Rachel Kimerling, Frank W. Weathers, Annabel Prins, Brian P. Marx, Edward P. Post, Paula P. Schnurr

**Affiliations:** 1 National Center for PTSD, VA Boston Healthcare System, Boston, Massachusetts; 2 Boston University School of Medicine, Boston, Massachusetts; 3 National Center for PTSD, VA Palo Alto Healthcare System, Palo Alto, California; 4 Center for Innovation to Implementation, VA Palo Alto Healthcare System, Palo Alto, California; 5 Department of Psychology, Auburn University, Auburn, Alabama; 6 Department of Psychology, San Jose State University, San Jose, California; 7 Veterans Affairs Central Office, Ann Arbor Veterans Affairs Healthcare System, Ann Arbor, Michigan; 8 University of Michigan Medical School, Ann Arbor; 9 National Center for PTSD, White River Junction, Vermont; 10 Geisel School of Medicine at Dartmouth, Hanover, New Hampshire

## Abstract

**Importance:**

Posttraumatic stress disorder (PTSD) is a serious mental health disorder that can be effectively treated with empirically based practices. PTSD screening is essential for identifying undetected cases and providing patients with appropriate care.

**Objective:**

To determine whether the Primary Care PTSD screen for the *Diagnostic and Statistical Manual of Mental Disorders* (Fifth Edition) (PC-PTSD-5) is a diagnostically accurate and acceptable measure for use in Veterans Affairs (VA) primary care clinics.

**Design, Setting, and Participants:**

This cross-sectional, diagnostic accuracy study enrolled participants from May 19, 2017, to September 26, 2018. Participants were recruited from primary care clinics across 2 VA Medical Centers. Session 1 was conducted in person, and session 2 was completed within 30 days via telephone. A consecutive sample of 1594 veterans, aged 18 years or older, who were scheduled for a primary care visit was recruited. Data analysis was performed from March 2019 to August 2020.

**Exposures:**

In session 1, participants completed a battery of questionnaires. In session 2, a research assistant administered the PC-PTSD-5 to participants, and then a clinician assessor blind to PC-PTSD-5 results conducted a structured diagnostic interview for PTSD.

**Main Outcomes and Measures:**

The range of PC-PTSD-5 cut points overall and across gender was assessed, and diagnostic performance was evaluated by calculating weighted κ values.

**Results:**

In total, 495 of 1594 veterans (31%) participated, and 396 completed all measures and were included in the analyses. Participants were demographically similar to the VA primary care population (mean [SD] age, 61.4 [15.5] years; age range, 21-93 years) and were predominantly male (333 participants [84.1%]) and White (296 of 394 participants [75.1%]). The PC-PTSD-5 had high levels of diagnostic accuracy for the overall sample (area under the receiver operating characteristic curve [AUC], 0.927; 95% CI, 0.896-0.959), men (AUC, 0.932; 95% CI, 0.894-0.969), and women (AUC, 0.899, 95% CI, 0.824-0.974). A cut point of 4 ideally balanced false negatives and false positives for the overall sample and for men. However, for women, this cut point resulted in high numbers of false negatives (6 veterans [33.3%]). A cut point of 3 fit better for women, despite increasing the number of false positives. Participants rated the PC-PTSD-5 as highly acceptable.

**Conclusions and Relevance:**

The PC-PTSD-5 is an accurate and acceptable screening tool for use in VA primary care settings. Because performance parameters will change according to sample, clinicians should consider sample characteristics and screening purposes when selecting a cut point.

## Key Points

**Question:**

Is the Primary Care Posttraumatic Stress Disorder (PTSD) Screen, as revised for the *Diagnostic and Statistical Manual of Mental Disorders* (Fifth Edition) (PC-PTSD-5), diagnostically accurate and acceptable to Veterans Affairs primary care patients?

**Findings:**

In this diagnostic study of 396 primary care–seeking veterans, the 5-item PC-PTSD-5 was both diagnostically accurate and acceptable to participants. A cut point score of 4 best balanced the false negatives and false positives, although women may require a lower cut point (ie, a score of 3).

**Meaning:**

Like its predecessor, the PC-PTSD-5 is an effective and efficient tool for PTSD screening in Veterans Affairs primary care clinics.

## Teaser

This diagnostic study examines whether the Primary Care Posttraumatic Stress Disorder (PTSD) screen for the *Diagnostic and Statistical Manual of Mental Disorders* (Fifth Edition) (*DSM-5*) is a diagnostically accurate and acceptable measure for use in Veterans Affairs primary care clinics.

## Introduction

Posttraumatic stress disorder (PTSD) is a serious mental health disorder that can develop following exposure to catastrophic life events, such as combat, sexual assault, and natural disasters. PTSD is associated with mental and physical health comorbidities,^1,2,3,4,5,6,7,8,9,10^ impaired functioning, and reduced quality of life.^11,12,13^ The lifetime prevalence of PTSD in the US is 6.1%.^14^ Among veterans who receive health care from the Department of Veterans Affairs (VA), the prevalence is 11.5% for men and 18.9% for women.^15,16^

To ensure identification of and appropriate services for individuals with PTSD, many health care systems, including VA, have instituted screening programs.^17,18^ In the VA system, all veterans enrolled in care are screened for PTSD annually for the first 5 years after military separation and every 5 years thereafter. In the VA system and in other settings, PTSD screening is conducted in primary care clinics^19,20^ because individuals with mental health symptoms are more likely to initially seek medical care.^21^ VA uses the Primary Care PTSD Screen (PC-PTSD)^22^ for screening. This 4-item measure, which is based on the PTSD diagnostic criteria in the *Diagnostic and Statistical Manual of Mental Disorders* (Fourth Edition),^23^ has excellent psychometric properties.^22,24,25,26^

In 2013, the American Psychiatric Association presented revised PTSD diagnostic criteria in the *Diagnostic and Statistical Manual of Mental Disorders* (Fifth Edition) (*DSM-5*).^27^ The PC-PTSD was revised as the PC-PTSD-5 to reflect *DSM-5* criteria. Furthermore, language to assess traumatic exposure was clarified to ensure that respondents understood that symptom questions referred to traumatic events and not to other stressors. Pilot testing suggested that the PC-PTSD-5 had sound diagnostic accuracy.^28,29^

The current study evaluates the PC-PTSD-5 using a reference-standard clinician-administered PTSD interview to establish the diagnostic accuracy and determine the appropriate cut point for use in a VA primary care setting. Because of gender differences in PTSD prevalence,^30^ we explore whether diagnostic accuracy and optimal PC-PTSD-5 cut point differed for men and women. Finally, we aim to confirm the high levels of patient-rated PC-PTSD-5 acceptability by replicating pilot findings.

## Methods

Local institutional review boards approved all study procedures. All participants provided written informed consent. This study follows the Standards for Reporting of Diagnostic Accuracy (STARD) reporting guideline^31^ (eAppendix 1 and eFigure in the Supplement).

### Participants

We approached a consecutive sample of primary care–seeking veterans across 2 VA Medical Centers. Veterans with complete data constituted our sample. Participants completed a number of measures, including a questionnaire that gathered information on demographic and military service characteristics.

### PC-PTSD-5 Screen

The PC-PTSD-5^29^ is a questionnaire designed to screen for PTSD in a primary care setting. Participants first complete a single question to determine lifetime trauma exposure. Those who deny exposure receive a score of 0. Participants who report exposure respond to 5 dichotomously scored (0 = no; 1 = yes) questions about PTSD symptoms experienced within the past month. Total scores range from 0 to 5.

### PC-PTSD-5 Acceptability Questionnaire

We modified a 7-item questionnaire to assess PC-PTSD-5 acceptability and participants’ preferred mode of administration (eAppendix 2 in the Supplement).^29^ All items were scored from 1 (very easy or very comfortable) to 5 (very difficult or very uncomfortable).

### Patient Health Questionnaire–9

The Patient Health Questionnaire–9 (PHQ-9)^32^ is a well-validated measure of *DSM-5* major depressive disorder.^33^ Respondents are asked to indicate how often they have been bothered by each symptom over the last 2 weeks on 9 items scored from 0 (not at all) to 3 (nearly every day). Consistent with VA practice, participants who screened positive on both the PHQ-2 (first 2 items; score ≥3) and the PHQ-9 (all 9 items; score ≥15) were considered positive for depression; participants with a score greater than or equal to 1 on the ninth item were considered to have screened positive for potential suicidality.

### Alcohol Use Disorders Identification Test–Consumption

The Alcohol Use Disorders Identification Test–Consumption (AUDIT-C) is a 3-item measure^34,35^ that detects *DSM-5* alcohol misuse disorders.^36^ Items are scored from 0 to 4, with higher scores indicating more use. Unlike the VA version of the measure, which asks about use in the past year, we used the validated version that does not specify the time frame of use. Consistent with VA practice, a score of 4 or higher for men and 3 or higher for women was considered a positive screen.

### Traumatic Brain Injury Screen

VA uses the 4-item Traumatic Brain Injury (TBI) Screen, which is based on the Brief Traumatic Brain Injury Screen,^37^ to identify veterans who were both exposed to events associated with TBI risk and who display symptoms potentially related to these exposures.^38^ Veterans who endorsed experiencing a TBI that resulted in symptoms both immediately after the event and within the past week were considered positive for TBI.

### Clinician-Administered PTSD Scale for *DSM-5*

The Clinician-Administered PTSD Scale for *DSM-5 *(CAPS-5)^39^ is the reference standard, clinician-rated diagnostic interview for the assessment of *DSM-5* PTSD. Each symptom is rated on a 5-point severity scale, ranging from 0 (absent) to 4 (extreme or incapacitating). Several scoring rules exist, ranging from broad to more stringent approaches to diagnosis.^40^ We used a moderately stringent approach, requiring symptoms to meet diagnostic criteria and a severity score of 22 or higher. This approach puts the diagnosis on the cusp of the mild-to-moderate range of PTSD^40^ and demonstrated the best fit to the data. (We calculated diagnostic accuracy statistics for additional scoring rules with nearly identical results; data not shown.) The CAPS-5 was administered by doctoral-level clinicians who participated in regular reliability meetings. Interrater reliability, which was calculated for 12.5% of the sample (51 participants), was excellent (κ = 0.88; intraclass correlation coefficient = 0.997).

### Procedure

From May 19, 2017, to September 26, 2018, veterans scheduled for a VA primary care visit who expressed interest in participation were scheduled for an in-person appointment on or within 7 days of their primary care visit. At this appointment (session 1), interested participants provided written consent and completed questionnaires. Participants were compensated $15 and were scheduled for a telephone appointment within 30 days of session 1 (session 2). The time between sessions ranged from 1 to 30 days (mean [SD], 11.9 [7.4] days). At session 2, participants first spoke to a research assistant who administered the PC-PTSD-5 and the PC-PTSD-5 Acceptability Questionnaire. The participant was then transferred to an assessor, blind to their PC-PTSD-5 results, who administered the CAPS-5. The quality and acceptability to research participants of psychiatric telephone interviews are now well-established.^41,42,43^ Veterans who participated in session 2 received an additional $30 compensation.

### Statistical Analysis

We conducted analyses to evaluate diagnostic accuracy and identify the optimal cut point for the overall sample and across gender. We examined diagnostic accuracy for each of the 5 possible cut points on the PC-PTSD-5 (1-5) against a CAPS-5 PTSD diagnosis (present/ vs absent) with weighted κ coefficients as measures of test quality, including quality of sensitivity (κ[1]), specificity (κ[0]), and efficiency (κ[0.5]). Quality metrics are measures of test performance (ie, sensitivity, specificity, and efficiency), that were calibrated for chance agreement between the test and diagnosis.^30^ We used a DAG_STAT spreadsheet^44^ to calculate measures of both test performance (for descriptive purposes) and test quality. Although optimally sensitive cut points are generally recommended for screening measures because they reduce false negatives, they also increase false positives.^30^ In contrast, optimally efficient cut points minimize diagnostic errors by decreasing false positives but increasing false negatives.^30^ Therefore, our objective was to compare the cut point with the highest quality of sensitivity that still maintained a minimal specificity (approximately 0.80; used to account for the cost of false positives) to the cut point with the highest quality of efficiency. The κ values were evaluated using the following guidelines: a value less than or equal to 0.40 is poor, a value greater than or equal to 0.41 and less than 0.60 is fair, a value greater than or equal to 0.60 and less than 0.75 is good, and a value greater than or equal to 0.75 is excellent.^45^

We also explored the potential impact of misclassified screens by examining the proportion of false negatives and false positives for each cut point that would receive a positive screen on other routine VA mental health screening instruments (AUDIT-C, PHQ-9, and TBI screen). If false-negative PTSD cases would come to clinical attention as a result of positive scores on other screens, concerns about underdetection of PTSD might be mitigated. If false-positive PTSD cases demonstrate clinically significant symptoms on other screens, concerns about excess diagnostic burden might be reduced, because these individuals would benefit from further evaluation.

We calculated descriptive statistics for the PC-PTSD-5 Acceptability Questionnaire and conducted paired *t* tests to determine the preferred mode of administration using SPSS statistical software version 26 (IBM). Significance was set at 2-sided *P* < .05. Data analysis was performed from March 2019 to August 2020.

## Results

We recruited 1594 primary care–seeking veterans across 2 VA Medical Centers and 495 (31%) participated. Complete data were available for 396 veterans, and they constituted our sample for analysis. Participants with and without complete data were demographically similar, with 3 exceptions: participants with complete data were more likely to be married (43.0% vs 16.7%; χ^2^ = 8.006; *P* = .005), less likely to be separated (2.0% vs 10.0%; χ^2^ = 7.033; *P* = .008), and less likely to be Latinx (ie, Latino or Latina; 7.8% vs 25.9%; χ^2^ = 10.898; *P* = .001) than those without complete data.

Participants ranged in age from 21 to 93 years (mean [SD], 61.4 [15.5] years) and were predominantly male (333 men [84.1%]), non-Latinx (320 of 347 participants [92.2%]), and White (296 of 394 participants [75.1%]). Eighty-eight participants (22.8%) served during the wars in Iraq and Afghanistan (ie, October 7, 2001, to present), and 298 participants (77.2%) served in an earlier era. Data were missing for 10 participants. See the eTable in the Supplement for participant characteristics by site.

According to the CAPS-5, 68 participants (17.2% of all participants, accounting for 50 men [15%] and 18 women [29%]) met the criteria for PTSD. Nearly all participants (374 participants [94.4%]) had experienced a traumatic event. The PC-PTSD-5 trauma item was generally concordant with the diagnostic interview, with 76.5% agreement (303 participants) on trauma exposure. Of the participants discordant for trauma exposure, only 1 (1.2%) met CAPS-5 PTSD criteria. Most discordant cases (86 of 93 participants [92.5%]) did not report exposure on the PC-PTSD-5 but trauma exposure was rated present upon clinical interview. These participants were significantly more likely to have experienced indirect exposure (learning about a traumatic event, rather than experiencing it directly) than participants who were concordant for exposure (χ^2^ = 8.28; *P* = .004).

### Diagnostic Accuracy

For the overall sample, the area under the receiver operating characteristic curve (AUC) for the PC-PTSD-5 was 0.927 (95% CI, 0.896-0.959). The cut point with the highest κ(1) value (0.85; excellent range) that still exceeded the a priori minimum specificity (≥0.80) on the PC-PTSD-5 was 3. With this cut point, 7 veterans (10.3%) with PTSD screened negative (false negatives), and 66 veterans (20.1%) without PTSD screened positive (false positives). The cut point with the highest κ(0.5) score (0.65; good range) was 4. With this cut point, the percentage of false negatives increased to 22.1% (15 false negatives), and the percentage of false positives decreased to 8.5% (28 false positives) (Table 1).

**Table 1.** Diagnostic Accuracy of PC-PTSD-5 for Estimating a CAPS-5 PTSD Diagnosis for the Overall Sample^a^

PC-PTSD-5 score cut point	Veterans, No. (%)			Sensitivity	Specificity	Efficiency	NPV	PPV	Positive LR	Negative LR	κ(0)^d^	κ(0.5)^e^	κ(1)^f^
	With PTSD	FN^b^	FP^c^										
1	212 (53.5)	1 (1.5)	145 (44.2)	.99	.56	.63	.99	.32	2.23	.03	.17	.30	.97
2	172 (43.4)	3 (4.4)	107 (32.6)	.96	.67	.72	.99	.38	2.93	.07	.25	.39	.92
3	127 (32.1)	7 (10.3)	66 (20.1)	.90	.80	.82	.97	.48	4.46	.13	.37	.52	.85
4	81 (20.5)	15 (22.1)	28 (8.5)	.78	.91	.89	.95	.65	9.13	.24	.58	.65	.72
5	41 (10.4)	33 (48.5)	6 (1.8)	.51	.98	.90	.91	.85	28.14	.49	.82	.59	.46

Abbreviations: CAPS-5, Clinician-Administered Posttraumatic Stress Disorder Scale for *Diagnostic and Statistical Manual of Mental Disorders* (Fifth Edition); FN, false negative; FP, false positive; LR, likelihood ratio; NPV, negative predictive value; PC-PTSD-5, Primary Care Posttraumatic Stress Disorder for *Diagnostic and Statistical Manual of Mental Disorders* (Fifth Edition); PPV, positive predictive value; PTSD, posttraumatic stress disorder.

^a^ CAPS-5 PTSD base rate is 17.2% (68 veterans).

^b^ Refers to veterans with CAPS-5 PTSD who did not screen positive on the PC-PTSD-5.

^c^ Refers to veterans without CAPS-5 PTSD who did screen positive on the PC-PTSD-5.

^d^ Refers to quality of specificity.

^e^ Refers to quality of efficiency.

^f^ Refers to quality of sensitivity.

For men, the AUC was 0.932 (95% CI, 0.894-0.969). The cut point with the highest κ(1) value (0.86; excellent range) that still demonstrated acceptable specificity (≥0.80) was 3. With this cut point, 5 male veterans (10.0%) were classified as false negatives, and 52 (18.4%) were classified as false positives. The cut point with the highest κ(0.5) value (0.67; good range) was 4. With this cut point, the percentage of false negatives increased to 18.0% (9 false negatives), whereas the percentage of false positives decreased to 7.8% (22 false positives) (Table 2).

**Table 2.** Diagnostic Accuracy of PC-PTSD-5 for Estimating a CAPS-5 PTSD Diagnosis for Male Veterans^a^

PC-PTSD-5 score cut point	Veterans, No. (%)			Sensitivity	Specificity	Efficiency	NPV	PPV	Positive LR	Negative LR	κ(0)^d^	κ(0.5)^e^	κ(1)^f^
	With PTSD	FN^b^	FP^c^										
1	167 (50.2)	1 (2.0)	118 (41.7)	.98	.58	.64	.99	.29	2.35	.03	.17	.29	.96
2	133 (40.0)	3 (6.0)	86 (30.4)	.94	.70	.73	.99	.35	3.09	.09	.24	.38	.90
3	97 (29.1)	5 (10.0)	52 (18.4)	.90	.82	.83	.98	.46	4.90	.12	.37	.52	.86
4	63 (18.9)	9 (18.0)	22 (7.8)	.82	.92	.91	.97	.65	10.55	.20	.59	.67	.78
5	30 (9.0)	25 (50.0)	5 (1.8)	.50	.98	.91	.92	.83	28.30	.51	.80	.58	.45

Abbreviations: CAPS-5, Clinician Administered Posttraumatic Stress Disorder Scale for *Diagnostic and Statistical Manual of Mental Disorders* (Fifth Edition); FN, false negative; FP, false positive; LR, likelihood ratio; NPV, negative predictive value; PC-PTSD-5, Primary Care Posttraumatic Stress Disorder for *Diagnostic and Statistical Manual of Mental Disorders* (Fifth Edition); PPV, positive predictive value; PTSD, posttraumatic stress disorder.

^a^ CAPS-5 PTSD base rate is 15.0% (50 veterans).

^b^ Refers to veterans with CAPS-5 PTSD who did not screen positive on the PC-PTSD-5.

^c^ Refers to veterans without CAPS-5 PTSD who did screen positive on the PC-PTSD-5.

^d^ Refers to quality of specificity.

^e^ Refers to quality of efficiency.

^f^ Refers to quality of sensitivity.

For women, the AUC was 0.899 (95% CI, 0.824-0.974). The AUC for men and women was not significantly different (χ^2^ = 0.57; *P* = .45), indicating that the PC-PTSD-5 performed consistently across gender. The cut point with the highest κ(1) value (0.79; excellent range) that still demonstrated acceptable specificity (≥0.80) was 3. With this cut point, 2 women (11.1%) were classified as false negatives, whereas 13 (30.0%) were classified as false positives. Unlike the overall sample and the male veterans, the cut point with the highest κ(0.5) value (0.60; good range) was 5. With this cut point, nearly half of female veterans (8 veterans [44.4%]) were classified as false negatives, whereas the percentage of false positives decreased to 2.3% (1 female veteran). Because a cut point of 5 would produce unacceptably high percentages of false negatives for both men and women, we also considered a cut point of 4. This cut point, which produced the second highest κ(0.5) value among women (0.56; fair range), tripled the number of false negatives associated with a cut point of 3 (6 women [33.3%]), but reduced the number of false positives by more than half (5 women [11.4%]) (Table 3).

**Table 3.** Diagnostic Accuracy of PC-PTSD-5 for Estimating a CAPS-5 PTSD Diagnosis for Female Veterans^a^

PC-PTSD-5 score cut point	Veterans, No. (%)			Sensitivity	Specificity	Efficiency	NPV	PPV	Positive LR	Negative LR	κ(0)^d^	κ(0.5)^e^	κ(1)^f^
	With PTSD	FN^b^	FP^c^										
1	44 (71.0)	0	26 (59.1)	1.00	.41	.58	1.00	.41	1.69	.00	.17	.29	1.00
2	38 (61.3)	0	20 (45.4)	1.00	.55	.68	1.00	.47	2.20	.00	.26	.41	1.00
3	29 (46.8)	2 (11.1)	13 (30.0)	.89	.70	.76	.94	.55	3.01	.16	.37	.50	.79
4	17 (27.4)	6 (33.3)	5 (11.4)	.67	.89	.82	.87	.71	5.87	.38	.59	.56	.54
5	11 (17.7)	8 (44.4)	1 (2.3)	.56	.98	.85	.84	.91	24.44	.45	.87	.60	.46

Abbreviations: CAPS-5, Clinician Administered Posttraumatic Stress Disorder Scale for *Diagnostic and Statistical Manual of Mental Disorders* (Fifth Edition); FN, false negative; FP, false positive; LR, likelihood ratio; NPV, negative predictive value; PC-PTSD-5, Primary Care Posttraumatic Stress Disorder for *Diagnostic and Statistical Manual of Mental Disorders* (Fifth Edition); PPV, positive predictive value; PTSD, posttraumatic stress disorder.

^a^ CAPS-5 PTSD base rate is 29.0% (18 veterans).

^b^ Refers to veterans with CAPS-5 PTSD who did not screen positive on the PC-PTSD-5.

^c^ Refers to veterans without CAPS-5 PTSD who did screen positive on the PC-PTSD-5.

^d^ Refers to quality of specificity.

^e^ Refers to quality of efficiency.

^f^ Refers to quality of sensitivity.

We conducted exploratory analyses to estimate the consequences of misclassification of PTSD status by the PC-PTSD-5. For PC-PTSD-5 cut points of 3 and 4, we examined the hypothetical likelihood that false-negative cases would come to clinical attention because of other mental health screens routinely administered in VA primary care. We also examined the likelihood that false-positive cases on the PC-PTSD-5 might benefit from diagnostic evaluation despite not meeting PTSD criteria, by determining whether other screens suggested clinically significant symptoms of other conditions. Results indicated that even with a cut point of 4, the cut point associated with the higher number of false negatives, most false negatives (7 men [77.8%]; 5 women [83.3%]) would otherwise come to clinical attention because of dispositions on 1 or more other screening instruments. Similarly, even with a cut point of 3, the cut point associated with the higher number of false positives, most false positives (44 men [84.6%]; 9 women [69.2%]) would likely benefit from additional evaluation, because they reported clinically significant symptoms of 1 or more other conditions (Table 4).

**Table 4.** Frequency of Positive Results for Other Routine Mental Health Screens in VA Primary Care Among Male and Female Veterans Misclassified by the PC-PTSD-5

Gender, screening instrument	Veterans, No. (%)			
	False negatives, PC-PTSD-5 score cut point		False positives, PC-PTSD-5 score cut point	
	3	4	3	4
Male				
Both PHQ-2 and PHQ-9	4 (80.0)	5 (55.6)	12 (23.1)	8 (36.4)
AUDIT-C	3 (60.0)	5 (55.6)	23 (44.2)	7 (31.8)
TBI screen	2 (40.0)	2 (22.2)	33 (63.5)	16 (72.7)
Ninth item of the PHQ-9	3 (60.0)	4 (44.4)	13 (25.0)	10 (45.5)
≥1 VA screen	5 (100.0)	7 (77.8)	44 (84.6)	22 (100.0)
Female				
Both PHQ-2 and PHQ-9	1 (50.0)	2 (33.3)	1 (8.3)^a^	0
AUDIT-C	1 (50.0)	3 (50.0)	3 (23.1)	1 (20.0)
TBI screen	2 (100.0)	4 (66.7)	7 (53.8)	3 (60.0)
Ninth item of the PHQ-9	1 (50.0)	3 (50.0)	2 (15.4)	1 (20.0)
≥1 VA screen	2 (100.0)	5 (83.3)	9 (69.2)	3 (60.0)

Abbreviations: AUDIT-C, Alcohol Use Disorders Identification Test–Consumption; PC-PTSD-5, Primary Care Posttraumatic Stress Disorder for *Diagnostic and Statistical Manual of Mental Disorders* (Fifth Edition); PHQ-2, Patient Health Questionnaire–2; PHQ-9, Patient Health Questionnaire–9; TBI, traumatic brain injury; VA, Department of Veterans Affairs.

^a^ One participant had missing data and was excluded from this analysis.

### Acceptability

Most participants rated the PC-PTSD-5 questions as easy or very easy to understand (372 participants [93.9%]) and answer (331 participants [83.6%]) and rated the instructions as clear or very clear (384 participants [97.0%]). Participants also indicated that they would be comfortable or very comfortable answering questions during a primary care visit (332 participants [83.8%]). Veterans were more comfortable answering the questions when asked by their own physician than either on their own (*t* = 2.76; *P* = .006) or by a nurse or other practitioner (*t* = −9.05; *P* < .001). Furthermore, veterans reported they would be more comfortable completing the PC-PTSD-5 on their own than with a nurse or other practitioner (*t* = −4.63; *P* < .001). There were no differences in administration preference across gender (all *t* < 1.39; all *P* > .15).

## Discussion

To our knowledge, this study is the first to examine the accuracy of the PC-PTSD-5 against a well-validated, clinician-administered structured interview. The PC-PTSD-5 had high levels of diagnostic accuracy. Weighted κ values indicated that the PC-PTSD-5 generally has good-to-excellent agreement with clinician-rated diagnosis. Furthermore, the inclusion of a trauma prompt added clarity to the screen; nearly all individuals for whom the screen failed to detect trauma exposure did not meet criteria for PTSD. Overall, the PC-PTSD-5 was well understood, and most participants felt comfortable completing it, with a preference for administration by the veteran’s own primary care practitioner. Results indicate that the PC-PTSD-5 retains the strong psychometric properties of its predecessor, the PC-PTSD.^22^ These findings are consistent with preliminary work examining the validity of the instrument^29^ and suggest that the PC-PTSD-5 can accurately detect PTSD in VA primary care settings.

The cut point on the PC-PTSD-5 with the highest quality of sensitivity given a minimal specificity was 3, whereas the cut point with the highest quality of efficiency was 4. Compared with a cut point of 4, a cut point of 3 was less likely to miss individuals with PTSD, but more likely to classify individuals without PTSD as having PTSD. These cut point options pose a choice between allocating resources toward detection vs the efficiency of clinical operations. Optimizing sensitivity will detect more cases of PTSD but could yield large numbers of primary care users who require further evaluation, many of whom will not receive a diagnosis of PTSD. Optimizing efficiency may mitigate the demand for diagnostic evaluation, especially among individuals without PTSD, but could fail to bring significant numbers of PTSD cases to the attention of practitioners.

For men, we again found that the cut point with the highest quality of sensitivity given a minimal specificity was 3, whereas the cut point with the highest quality of efficiency was 4; because men made up 84.3% of the sample, this parallel is unsurprising. For women, the cut point with the highest quality of sensitivity given a minimal specificity was also 3, but the cut point with the highest quality of efficiency was 5. The differential findings across gender may be driven by differences in PTSD prevalence: PTSD was nearly twice as prevalent in women vs men. Although the AUC did not differ by gender, stratified analyses suggested that, at a cut point of 4, there is risk of a substantial proportion of undetected PTSD among women, without the similar reduction of false positives observed among men. Additional research with larger populations of women can inform judgments regarding the utility of lower cut points among women in some settings.

False-positive screens risk excess diagnostic burden that may limit the efficiency of clinical systems, but the cost of undetected PTSD could be even more substantial in terms of the avoidable suffering, health risks, and increased health care costs associated with untreated PTSD.^13,46,47^ Our exploratory analyses illustrate the likely disposition of undetected cases under current VA mental health screening practice: the majority of undetected PTSD cases would likely come to clinical attention for evaluation as a result of their disposition from other routine mental health screens in primary care. To the extent that these clinical encounters lead to the detection of PTSD and/or treatment of comorbid conditions, the costs of undetected PTSD would be mitigated.

### Limitations

Our study has several limitations. First, participants were veterans in the VA health care system, which may limit generalizability to other health care settings or populations. However, we expect that, like the PC-PTSD, the PC-PTSD-5 will retain its strong psychometric properties across nonveteran samples.^20,24,26^ Second, although participants were demographically representative of VA primary care users,^48,49^ nonresponse bias may limit generalizability to this population. Third, other subgroups beyond and within gender may benefit from further study; this is an important future direction. Fourth, our interpretation of the ratio of harms to benefits for different cut points is predicated on the assumption that VA primary care–based mental health screens are administered within similar time frames. If they are not, the proportion of undetected cases that ultimately receive clinical attention may differ from our estimates. Future research is encouraged to replicate findings in a naturalistic setting to evaluate these estimates.

## Conclusions

Overall, the PC-PTSD-5 is a useful PTSD screen that is easily understood and completed by primary care–seeking veterans and demonstrates good diagnostic utility. However, like all screens, the PC-PTSD-5 is not a substitute for clinical judgment. Scores of 3 among individuals presenting with symptoms of PTSD may merit diagnostic evaluation despite their screen scores. Practitioners may consider a lower cut point for female veterans in some settings if evaluation resources are available or base rates of mental health conditions are high. In contrast, a higher cut point may be preferable if resources are such that false positives will substantially decrease clinician availability. Because performance parameters will change according to sample, clinicians should consider sample characteristics and screening purposes when selecting a cut point.

Regardless of the cut point, the PC-PTSD-5 has demonstrated utility in screening for PTSD in VA primary care settings. The prevalence of PTSD among patients seeking primary care—15% of men and 29% of women in our sample—underscores the importance of screening. PTSD is a treatable condition, with numerous treatment options.^18^ Screening is an essential step toward getting patients the care they need.

## Notes

Supplement. **eAppendix 1.** Information on Power Calculations **eFigure.** Standards for the Reporting of Diagnostic Accuracy Studies (STARD) Diagram of Patient Flow through the Study **eAppendix 2.** PC-PTSD-5 Acceptability Questionnaire **eTable.** Comparison of Recruitment Sites
