# Supplementary material for: Diagnostic Accuracy and Acceptability of the Primary Care Posttraumatic Stress Disorder Screen for the Diagnostic and Statistical Manual of Mental Disorders (Fifth Edition) Among US Veterans
Source: JAMA Netw Open. 2021 Feb 4;4(2):e2036733. doi: 10.1001/jamanetworkopen.2020.36733 (PMC7862990; doi:10.1001/jamanetworkopen.2020.36733)
Supplement: Supplement. — eAppendix 1. Information on Power Calculations eFigure. Standards for the Reporting of Diagnostic Accuracy Studies (STARD) Diagram of Patient Flow through the Study eAppendix 2. PC-PTSD-5 Acceptability Questionnaire eTable. Comparison of Recruitment Sites [file jamanetwopen-e2036733-s001.pdf]

Supplemental Online Content

Bovin MJ, Kimerling R, Weathers FW, et al. Diagnostic accuracy and acceptability of the primary care posttraumatic stress disorder screen for the *Diagnostic and Statistical Manual of Mental Disorders* (Fifth Edition) among US veterans. *JAMA Netw Open*. 2021;4(2):e2036733. doi:10.1001/jamanetworkopen.2020.36733

eAppendix 1. Information on Power Calculations

eFigure. Standards for the Reporting of Diagnostic Accuracy Studies (STARD) Diagram of Patient Flow through the Study

eAppendix 2. PC-PTSD-5 Acceptability Questionnaire

eTable. Comparison of Recruitment Sites

This supplemental material has been provided by the authors to give readers additional information about their work.

© 2021 Bovin MJ et al. *JAMA Network Open.*

eAppendix 1. Information on Power Calculations

Our aim of determining the optimal cutoff score for the PC-PTSD-5 is not based on inferential statistics. Because determination of a cutoff score does not use an inferential test, traditional inferential statistical methods do not apply to these analyses. However, heuristics developed to guide power estimates for diagnostic utility work were used to guide sample size projections. Specifically, Kraemer (1992) recommended that studies involving kappa should have at least 10 cases in each marginal (i.e., in our case, PC-PTSD-5 positive, PC-PTSD-5 negative, CAPS-5 positive, CAPS-5 negative). Therefore, we aimed to collect a dataset of approximately 400 participants, estimating conservatively that prevalence would be 10% for the overall sample and for men, and 15% for women. Based on the higher rates of PTSD identified in our sample, this heuristic suggests that we are not only adequately powered for analyses using the full sample (prevalence = 17.2%; sample size needed = 67; sample size achieved = 396), but also for the sample of men (prevalence = 15.0%; sample size needed = 59; sample size achieved = 333) and women (prevalence = 29.0%; sample size needed = 35; sample size achieved = 62).

© 2021 Bovin MJ et al. *JAMA Network Open.*

eFigure. Standards for the Reporting of Diagnostic Accuracy Studies (STARD) Diagram of Patient Flow Through the Study

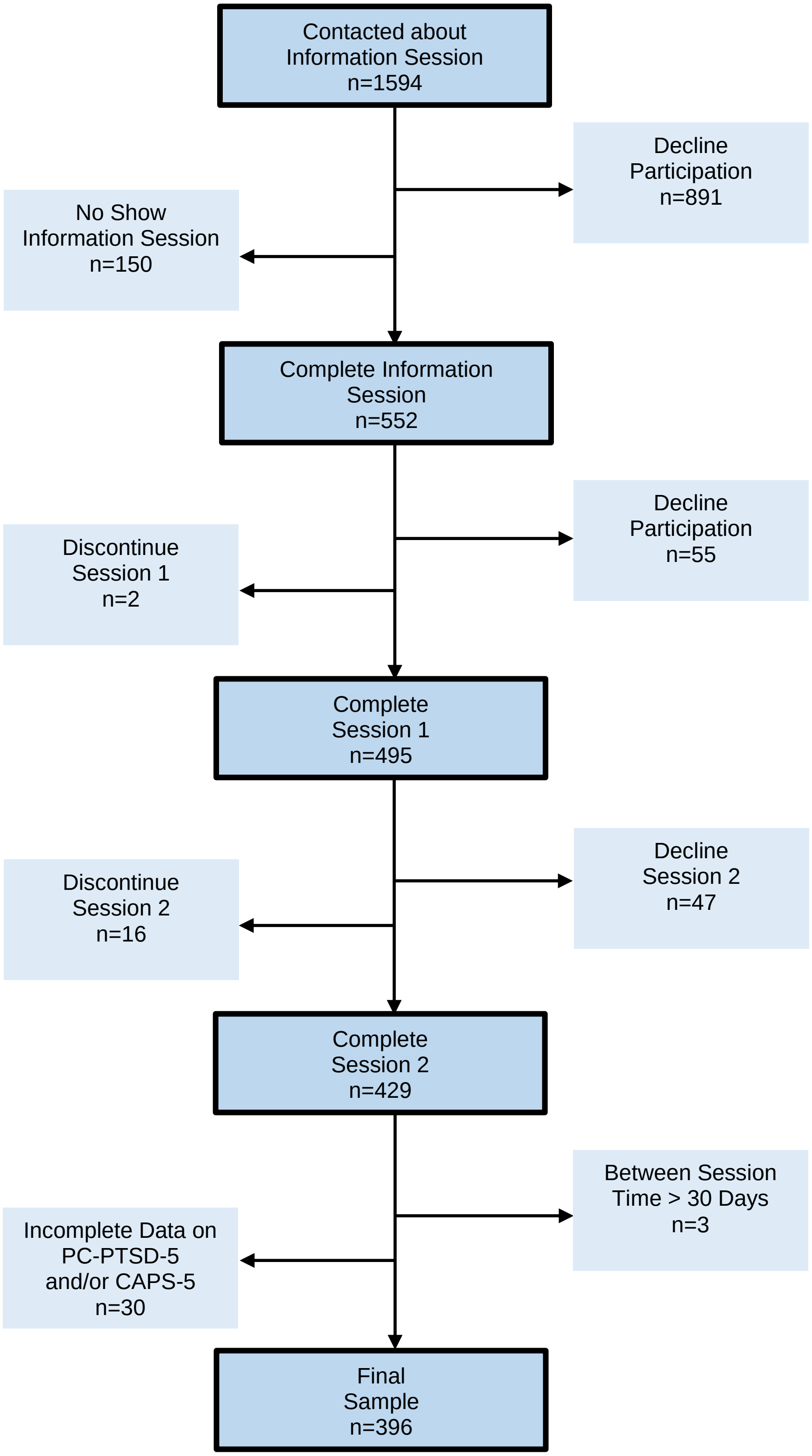

© 2021 Bovin MJ et al. *JAMA Network Open.*

eAppendix 2. PC-PTSD-5 Acceptability Questionnaire

Now I would like you to think back on the last (few) question(s) about stressful and traumatic events.

How easy was it for you to understand the(se) question(s)?

1) Very easy
2) easy
3) not sure
4) difficult
5) Very difficult

How easy was it for you to answer the(se) question(s)?

1) Very easy
2) easy
3) not sure
4) difficult
5) Very difficult

How clear were the instructions about how to answer the(se) question(s)?

1) Very clear
2) clear
3) not sure
4) unclear
5) Very unclear

How comfortable would you be answering the(se) question(s) at a visit to your primary care doctor?

1) Very comfortable
2) comfortable
3) not sure
4) uncomfortable
5) Very uncomfortable

How comfortable would you be in answering the(se) question(s), if you are asked to fill out a paper or electronic form on your own?

1) Very comfortable
2) comfortable
3) not sure
4) uncomfortable
5) Very uncomfortable

How comfortable would you be in answering the(se) questions, if you are asked by your doctor?

1) Very comfortable

© 2021 Bovin MJ et al. *JAMA Network Open.*

2) comfortable
3) not sure
4) uncomfortable
5) Very uncomfortable

How comfortable would you be in answering the(se) questions, if you are asked by a nurse or other providers?

1) Very comfortable
2) comfortable
3) not sure
4) uncomfortable
5) Very uncomfortable

© 2021 Bovin MJ et al. *JAMA Network Open.*

eTable. Comparison of Recruitment Sites

	**Site 1 (*n* = 214)**	**Site 2 (*n* = 182)**	***t*/χ^2**
**Age (*M, SD*)**	60.0 (15.8)	63.0 (14.9)	1.92
**Gender % (n) Female**	16.4 (35)	14.8 (27)	.19
**Race % (*n*)**			
**White**	76.4 (162)	73.6 (134)	.41
**Black**	19.3 (41)	14.3 (26)	1.77
**Asian**	0.9 (2)	7.7 (14)	11.45*
**Native American**	0.9 (2)	5.5 (10)	6.87*
**Pacific Islander**	0.0 (0)	3.3 (6)	7.10*
**Other**	4.7 (10)	6.0 (11)	.34
**Ethnicity % (*n*)**			
**Latinx**	4.4 (9)	12.8 (18)	8.23*
**Marital Status % (*n*)**			
**Never married**	23.5 (50)	17.0 (31)	2.50
**Living with partner**	4.7 (10)	1.6 (3)	2.86
**Married**	39.9 (85)	46.7 (85)	1.85
**Separated**	1.9 (4)	2.2 (4)	.05
**Divorced**	26.3 (56)	29.1 (53)	.39
**Widowed**	6.6 (14)	4.4 (8)	.89

* = Sites differ from one another at the $p < .05$ level

© 2021 Bovin MJ et al. *JAMA Network Open.*
